# Complete genome sequence of “*Candidatus* Dehalogenimonas loeffleri” strain W, a highly salt-tolerant chlorinated alkane-dechlorinating bacterium isolated from estuarine sediments

**DOI:** 10.1128/mra.00319-24

**Published:** 2024-11-27

**Authors:** Hongyan Wang, Xin Wang, Siqi Huang, Shujing Yang, Hengyi Liao, Xuhao Wang, Huijuan Jin, Jingjing Wang, Xiuying Li, Jun Yan, Yi Yang

**Affiliations:** 1 Key Laboratory of Pollution Ecology and Environmental Engineering, Institute of Applied Ecology, Chinese Academy of Sciences, Shenyang, Liaoning, China; 2 University of Chinese Academy of Sciences, Beijing, China; 3 School of Pharmaceutical Engineering, Shenyang Pharmaceutical University, Shenyang, Liaoning, China; 4 Key Laboratory of Forest Ecology and Silviculture, Institute of Applied Ecology, Chinese Academy of Sciences, Shenyang, Liaoning, China; California State University San Marcos, San Marcos, California, USA

**Keywords:** *Dehalogenimonas*, salt-tolerant, estuarine sediment, reductive dechlorination, organohalide-respiring bacteria, 1,2-dichloroethane, dihaloelimination

## Abstract

*“Candidatus* Dehalogenimonas loeffleri” strain W, isolated from estuarine sediments, can dechlorinate 1,2-dichloroethane under high salinity. Its genome consists of a circular 1,772,240-bp chromosome with a G + C content of 52.5% and encompasses 1,763 protein-coding sequences, including 28 genes encoding reductive dehalogenases.

## ANNOUNCEMENT

Bioremediation of chlorinated contaminants in saline environments is challenging due to the inability of most organohalide-respiring bacteria (OHRB) to thrive under elevated salinity conditions (1–4), highlighting our limited understanding of OHRB diversity and survival mechanisms in saline habitats. To date, only four species within *Dehalogenimonas* have been identified, with a variety of isolates originating from diverse terrestrial habitats (2–5). “*Candidatus* Dehalogenimonas loeffleri” strain W exhibits 1,2-dichloroethane (1,2-DCA) dechlorinating activity under high salinity conditions (e.g., 5.1% NaCl), a trait that has not been previously reported in *Dehalogenimonas* species. Strain W was enriched from estuarine sediments collected in June 2018 where the River Wuli meets the Bohai Sea in Huludao City, Liaoning Province, China (40°44′44″N, 120°59′11″E), and isolated using a dilution-to-extinction approach in bicarbonate-buffered (30 mM) basal mineral salt medium (6), supplemented with 1 g liter^−1^ ampicillin, 0.1 g liter^−1^ vancomycin, 5 mM acetate (carbon source), 413 µmol hydrogen (electron donor), 1,2-DCA (56.3 µmol, electron acceptor), and Wolin vitamin mix. Incubation was at 30°C in the dark without agitation.

Genomic DNA from strain W was extracted using the cetyltrimethylammonium bromide method (7). For PacBio sequencing, genomic DNA was sheared into 10-kb fragments using g-TUBEs (Covaris, USA) to prepare a long-insert library without size selection. These fragments were then ligated with universal hairpin adapters using the SMRTbell Express Template Prep Kit version 2.0 (Pacific Biosciences, USA) (8). Sequencing was subsequently performed using the Sequel II System. Raw read quality control, error correction, and adapter trimming were performed using the SMRT Link version 10.1. For Illumina sequencing, a library with an average insert size of 350 bp was constructed using the NEBNext Ultra DNA Library Prep Kit (New England Biolabs, USA), followed by paired-end sequencing (2 × 150 bp) on an Illumina NovaSeq 6000 instrument (Illumina Inc., USA). The quality-control filtering was conducted using fastp version 0.23.4 (9) for short Illumina reads and Filtlong version 0.2.1 (10) for long PacBio reads, respectively. Assembly integrating PacBio-filtered long reads with a read *N*50 of 10,710 bp (coverage, 478×) and Illumina-filtered short reads (coverage, 852×) was accomplished using Unicycler version 0.47 (Table 1) (11). Sequencing data were analyzed on the Galaxy web platform (https://usegalaxy.org.au) (12). Default parameters were used unless otherwise noted. The complete genome of strain W, assembled as a single circular chromosome of 1,772,240 bp with a G + C content of 52.5%, was annotated using the NCBI Prokaryotic Genome Annotation Pipeline version 6.6 for gene prediction and functional assignment (13). This analysis revealed 1,763 predicted protein-coding genes, 47 tRNAs, and a single copy each of the 5S rRNA, 16S rRNA, and 23S rRNA genes. The genome harbors 28 nonidentical reductive dehalogenase (*rdh*) subunit A genes, including one encoding a putative dihaloelimination RDase (V8247_04055, DdeA) that shares a remarkable 91.4%–94.9% amino acid identity with DcpA, previously identified in *Dehalococcoides mccartyi* strains RC and KS (14), *Dehalogenimonas formicexedens* strain NSZ-14^T^ (3), and *Dehalogenimonas lykanthroporepellens* strain BL-DC-9^T^ (14). Average nucleotide identity (ANI) analysis using JSpeciesWS version 4.1.1 (15) demonstrated that strain W shares 69.1%–73.7% ANI with *Dehalogenimonas* strains BL-DC-9^T^ (CP002084.1), WBC-2 (CP011392.1), IP3-3^T^ (LFDV00000000.1), NSZ-14^T^ (CP018258.1), and GP^T^ (CP058566.2), suggesting that strain W may represent a new species within the genus *Dehalogenimonas*. The genome of strain W expands the *Dehalogenimonas* pangenome and provides a foundation for elucidating the molecular mechanisms underpinning its remarkable ability to perform reductive dechlorination under elevated salinity conditions.

**TABLE 1:** Total reads obtained from PacBio and Illumina sequencing

Sequencing type	Number of raw reads	Number of filtered reads	Coverage
PacBio	146,715	52,627	478×
Illumina	5,761,950	4,840,340	852×

## Data Availability

The complete genome sequence of "*Candidatus Dehalogenimonas loeffleri*" strain W has been deposited in DDBJ, ENA, and GenBank (accession number CP146612.1). The BioSample and BioProject accession numbers are SAMN40212058 and PRJNA1082492, respectively. Raw sequences have been deposited in the Sequence Read Archive (SRA) under accession numbers SRR30671311, SRR30671312, SRR30671313 (PacBio), and SRR28404127 (Illumina).

## References

[1] Asai M, Yoshida N, Kusakabe T, Ismaeil M, Nishiuchi T, Katayama A. 2022. Dehalococcoides mccartyi NIT01, a novel isolate, dechlorinates high concentrations of chloroethenes by expressing at least six different reductive dehalogenases. Environ Res 207:112150. doi:10.1016/j.envres.2021.112150 34619124

[2] Bowman KS, Nobre MF, da Costa MS, Rainey FA, Moe WM. 2013. Dehalogenimonas alkenigignens sp. nov., a chlorinated-alkane-dehalogenating bacterium isolated from groundwater. Int J Syst Evol Microbiol 63:1492–1498. doi:10.1099/ijs.0.045054-0 22888191

[3] Key TA, Bowman KS, Lee I, Chun J, Albuquerque L, da Costa MS, Rainey FA, Moe WM. 2017. Dehalogenimonas formicexedens sp. nov., a chlorinated alkane-respiring bacterium isolated from contaminated groundwater. Int J Syst Evol Microbiol 67:1366–1373. doi:10.1099/ijsem.0.001819 28126048

[4] Cui Y, Li X, Yan J, Lv Y, Jin H, Wang J, Chen G, Kara-Murdoch F, Yang Y, Löffler FE. 2023. Dehalogenimonas etheniformans sp. nov., a formate-oxidizing, organohalide-respiring bacterium isolated from grape pomace. Int J Syst Evol Microbiol 73. doi:10.1099/ijsem.0.005881 37185088

[5] Moe WM, Yan J, Nobre MF, da Costa MS, Rainey FA. 2009. Dehalogenimonas lykanthroporepellens gen. nov., sp. nov., a reductively dehalogenating bacterium isolated from chlorinated solvent-contaminated groundwater. Int J Syst Evol Microbiol 59:2692–2697. doi:10.1099/ijs.0.011502-0 19625421

[6] Löffler FE, Sanford RA, Ritalahti KM. 2005. Enrichment, cultivation, and detection of reductively dechlorinating bacteria. Method Enzymol 397:77–111. 10.1016/S0076-6879(05)97005-5 16260286

[7] Feil WS, Feil H, Copeland A. 2012. Bacterial genomic DNA isolation using CTAB. Available from: https://jgi.doe.gov/wp-content/uploads/2014/02/JGI-Bacterial-DNA-isolation-CTAB-Protocol-2012

[8] McCarthy A. 2010. Third generation DNA sequencing: pacific biosciences’ single molecule real time technology. Chem Biol 17:675–676. doi:10.1016/j.chembiol.2010.07.004 20659677

[9] Chen S, Zhou Y, Chen Y, Gu J. 2018. fastp: an ultra-fast all-in-one FASTQ preprocessor. Bioinformatics 34:i884–i890. doi:10.1093/bioinformatics/bty560 30423086 PMC6129281

[10] Wick RR, Menzel P. 2018. GitHub. Filtlong. Available from: https://github.com/rrwick/Filtlong

[11] Wick RR, Judd LM, Gorrie CL, Holt KE. 2017. Unicycler: resolving bacterial genome assemblies from short and long sequencing reads. PLoS Comput Biol 13:e1005595. doi:10.1371/journal.pcbi.1005595 28594827 PMC5481147

[12] Galaxy Community. 2022. The Galaxy platform for accessible, reproducible and collaborative biomedical analyses: 2022 update. Nucleic Acids Res 50:W345–W351. doi:10.1093/nar/gkac247 35446428 PMC9252830

[13] Tatusova T, DiCuccio M, Badretdin A, Chetvernin V, Nawrocki EP, Zaslavsky L, Lomsadze A, Pruitt KD, Borodovsky M, Ostell J. 2016. NCBI prokaryotic genome annotation pipeline. Nucleic Acids Res 44:6614–6624. doi:10.1093/nar/gkw569 27342282 PMC5001611

[14] Padilla-Crespo E, Yan J, Swift C, Wagner DD, Chourey K, Hettich RL, Ritalahti KM, Löffler FE. 2014. Identification and environmental distribution of dcpA, which encodes the reductive dehalogenase catalyzing the dichloroelimination of 1,2-dichloropropane to propene in organohalide-respiring chloroflexi. Appl Environ Microbiol 80:808–818. doi:10.1128/AEM.02927-13 24242248 PMC3911209

[15] Richter M, Rosselló-Móra R, Oliver Glöckner F, Peplies J. 2016. JSpeciesWS: a web server for prokaryotic species circumscription based on pairwise genome comparison. Bioinformatics 32:929–931. doi:10.1093/bioinformatics/btv681 26576653 PMC5939971

